# Case of a Scirrhous Tumour in the Breast, Cured by Dispersion

**Published:** 1802-01

**Authors:** James Lucas

**Affiliations:** Member of the Royal College of Surgeons, and corresponding Member of the London Medical Society


### *Case of a scirrhous Tumour in the Breast, cured by Dispersion.*

*Communicated to* JOHN PEARSON, Efq. *Surgeon of the Lock Hospital and Asylum, and of the Public Dispensary, &c. by* Mr. JAMES LUCAS, *Member of the Royal College of Surgeons, and corresponding Member of the London Medical Society.*

A. R. between thirty and forty years of age, of a corpulent habit, had an enlargement in her breaft, exceeding the fize of a turkey's egg. The tumour, which had been gradually increafing for a year, had lately made a more rapid progrefs, and become fo indurated and painful as to oblige her to apply for relief; nor could fhe affign any caufe for the complaint.

The application of leeches was directed to be repeated according to the urgency of the pain. A piece of linen, in feveral folds, moiftened with an anodyne compound of litharge

 lotion, lotion, was frequently applied to the ſwelling, and increaſed doſes of conium maculatum were preſcribed. When theſe remedies had been ſtrictly perſiſted in for ſeveral weeks, it was obſerved, that the liberal evacuations had conſtantly afforded relief, but that the patient was conſiderably reduced. The bulk of the tumour was evidently diminiſhed, yet the nipple continued to be much depreſſed, and the ſkin around it had become ſomewhat inflamed, nor could the induration be ſaid to be abated. She now complained more of foreneſs than the lancinating pains that had before created ſuch uneaſineſs.

The topical bleedings were ordered to be omitted, unleſs the pain ſhould be urgent. Applications of cold water were uſed inſtead of the former lotion.—Doſes of the ferrum ammoniacale were given twice or three times a day, and the conium maculatum at bed-time.—Such variations in the diet were directed as ſuited the changes in the medical treatment. In a few weeks the whole breaſt had a leſs malignant appearance, the diminution of the tumour was evidently progreſſive, and the pain entirely ceaſed. A ſoap cerate plaiſter was in a little time ordered for a ſmall remaining ſoft tumour, rather to be diſcovered by the touch than the eye.

I have wiſhed to tranſmit you this caſe, and could cite many that occurred in the courſe of my practice at Leeds, as according with your opinion on the ſubject. I have, as far as I could, abbreviated the circumſtances in order to ſubjoin a few remarks. The ſucceſs in treating ſuch caſes ſeems to depend on the age being favourable, in allowing a cautious ſurgeon to try ſuch means as have been found uſeful, and in keeping all expectation of the neceſſity of an operation out of the patient's or even her friends view, until the ſtage of the malady prompts the moſt ſuitable time for extirpation. Great diſcernment is required to make choice of the moſt proper period for the neceſſity of an operation. If adviſed before it be neceſſary, the effect will generally prove highly prejudicial to the patient's health, even for years, if not through life; or a relapſe of a more dangerous diſeaſe will enſue, ſo that a premature extirpation ought to be as carefully avoided, as delay at an advanced period of life, when the rapid progreſs is known to extend itſelf beyond hope of aſſiſtance, even by operation, except timely performed. I have ſtrong reaſons for doubting if an *ulcerated cancer* in a breaſt, or limb, was ever permanently cured by an operation, although the wound may have healed ſpeedily, and the cure appeared promiſing. In the lip I allow the cure will be more laſting. Were I allowed to enlarge on this topic I could bring forward numerous examples of ſcirrhous tumours in the breaſt being relieved, and the diſeaſe never proving fatal, or or even becoming ulcerated, though not entirely removed. The moſt ſtriking inſtance that occurs to my recollection, was a woman, who had at times been under a courſe of remedies for a tumour in her breaſt, from thirty to nearly ninety years of age, and though the ſkin then inflamed yet there was no ulcer, nor did ſhe die of that complaint.

*Coniſbrough, near Doncaſter, Dec.* 1, 1801.

